# Protocol for analyzing T cell and macrophage motility in a mouse pancreatic cancer spheroid model using light-sheet microscopy

**DOI:** 10.1016/j.xpro.2025.103981

**Published:** 2025-07-26

**Authors:** Vanessa Zimmer, Benedikt Slusny, Katrin Roth, Christian Bauer

**Affiliations:** 1 Department of Gastroenterology, Endocrinology, Infectious Diseases and Metabolism, Philipps University Marburg, 35043 Marburg, Germany; 2 Core Facility Cellular Imaging, Center for Tumor Biology and Immunology, Philipps University Marburg, 35043 Marburg, Germany; 3 Department of Gastroenterology, DonauIsar Klinikum Deggendorf, MedizinCampus Niederbayern, 94469 Deggendorf, Germany

**Keywords:** Cell culture, Immunology, Microscopy

## Abstract

The interaction of macrophages and T cells is pivotal for shaping the immune response to cancer. Here, we present a protocol for investigating the co-culture of macrophages and T cells in a pancreatic cancer spheroid model. We describe steps for analyzing migration patterns by light-sheet microscopy, generating murine bone marrow-derived macrophages (BMDMs) and cytotoxic T lymphocytes (CTLs), and staining CTLs for image analysis. We then detail procedures for generating spheroids using a mouse pancreatic cancer cell line.

For complete details on the use and execution of this protocol, please refer to Slusny et al.^1^

## Highlights

• Co-culture of BM-derived macrophages and T cells in a pancreatic cancer spheroid model
• Detailed instructions for the formation of murine pancreatic cancer spheroids
• Time-lapse imaging of T cells and macrophages using light-sheet microscopy

## Abstract (editor highlights)

Publisher’s note: Undertaking any experimental protocol requires adherence to local institutional guidelines for laboratory safety and ethics.

## Graphical abstract

**Figure:**
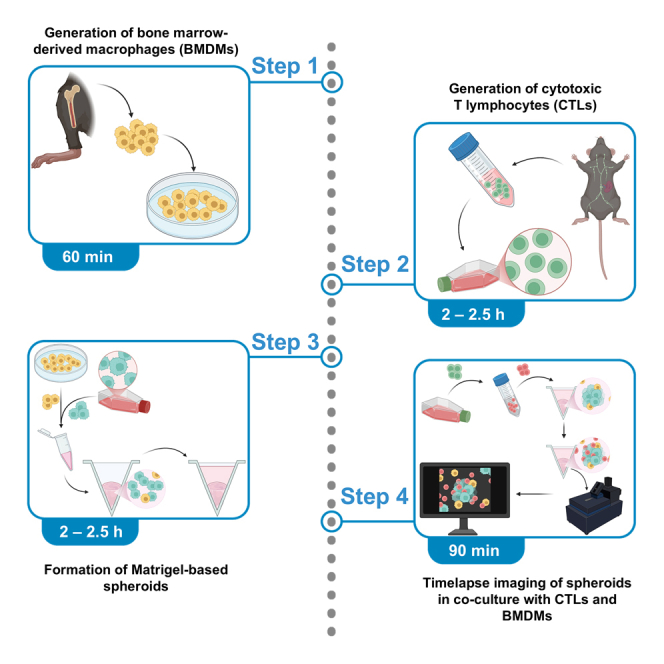

## Before you begin

### Institutional permissions

Acquire institutional permission for animal experimentation from the respective institution.

All animal experiments were conducted strictly following guidelines for handling experimental animals and were approved by the animal welfare advisor of Philipps University Marburg, Germany.

### Preparation of surgical tools

**Timing: 20 min**

1. To remove the femur, tibia, spleen, and lymph nodes, use clean and sterile pairs of scissors and forceps. Working in a sterile setting is mandatory and requires autoclaving of instruments.

### Preparation of cell culture media

**Timing: 10 min**

The exact composition of all media can be found in “materials and equipment”.

2. T cell/BMDM medium.
  a. For 500 mL T cell/BMDM medium, mix 415 mL RPMI 1640 + L-glutamine with 50 mL fetal calf serum (FCS), 15 mL 2-[4-(2-Hydroxyethyl)-1-piperazinyl]-ethane sulfonic acid (HEPES; 1 M), 10 mL Penicillin/Streptomycin solution (Pen/Strep; 10.00 U/mL/10.000 μg/mL), 10 mL sodium pyruvate (NaPyr; 100 mM), and 500 μL 2-mercaptoethanol.
  b. Store at 4°C for up to 8 weeks.
  c. Before use, heat to 37°C in a water bath.
3. PancOVA medium.
  a. For 500 mL PancOVA medium, mix 445 mL DMEM with 50 mL FCS and 5 mL Pen/Strep (10.000 U/mL/10.000 μg/mL).
  b. Store at 4°C for up to 8 weeks.
  c. Before use, heat to 37°C in a water bath.
  d. Add 500 μg/mL G418 disulfate solution (stock: 50 mg/mL) to the medium for positive selection of PancOVA cells.

### Preparation of erythrocyte lysis buffer

**Timing: 5 min**

The exact composition can be found in “materials and equipment”.

4. For the preparation of 500 mL erythrocyte lysis buffer.
  a. Add 155 mM NH_4_Cl, 10 mM KCO_3_, and 0.1 mM EDTA to 500 mL deionized H_2_O (dH_2_O).
  b. Sterile filter the solution with a sterile disposable filtration device.
  c. Store at 4°C for up to 6 months.

### Preparation of mice

Mice of the lipid-modified YFP transgenic TgVenus (Tg(CAG-Venus)1Hadj/J; JAX:011107) strain were used for the generation of BMDMs.

Spleen and lymph nodes from OT-I transgenic T cell receptor (C57BL/6-Tg(TcraTcrb)1100Mjb/J; JAX:003831) mice were used to generate CTLs.

All mice had a C57BL/6J background and were 8–16 weeks old. Both female and male mice were used.

***Optional:*** T cells from mice carrying more than one transgene can be used, e.g., OT-Ix*Il18r*^−/−^ T cells. OT-I mice were mated with B6.129P2-*Il18rltm1Aki*/J (JAX: 004131) to generate a mouse strain with transgenic T cell receptor and IL-18 receptor deficiency. Both female and male mice of 8–16 weeks of age were used, as described.^1^^,^^2^

## Key resources table

**Table:** 

REAGENT or RESOURCE	SOURCE	IDENTIFIER
**Chemicals, peptides, and recombinant proteins**		

2-mercaptoethanol	Merck	Cat#805740
Accutase	Sigma-Aldrich	A6964-100ML
CellTracker Deep Red Dye	Thermo Fisher Scientific	Cat#C34565
DMEM	Thermo Fisher Scientific	Cat#41965-062
Ethylenediaminetetraacetic acid (EDTA)	Sigma-Aldrich	E1644-100G
FCS	Capricorn Scientific	Cat#FCS-11A
G418 disulfate solution (Geneticin, 50 mg/mL)	Sigma-Aldrich	G8168-10ML
HBSS	Thermo Fisher Scientific	Cat#15420-614
HEPES (1 M)	Thermo Fisher Scientific	Cat#15360-056
Recombinant mouse IFN-γ, dissolved in PBS + 0.1% BSA to a concentration of 10 μg/mL	Peprotech	Cat#315-05
Recombinant mouse IL-2, dissolved in PBS + 0.1% BSA to a concentration of 20 μg/mL	Peprotech	Cat#212-12
Recombinant mouse IL-4, dissolved in PBS + 0.1% BSA to a concentration of 20 μg/mL	Peprotech	Cat#214-14
Recombinant mouse IL-12, dissolved in PBS + 0.1% BSA to a concentration of 10 μg/mL	Peprotech	Cat#210-12
Potassium carbonate (KCO_3_)	Carl Roth	Cat#6781.1
Lipopolysaccharides (LPS) from *E.coli* O127:B8	Sigma-Aldrich	L3129-10MG
Matrigel, growth factor reduced	Corning	Cat#356231
Recombinant mouse M-CSF, dissolved in PBS + 0.1% BSA to a concentration of 50 μg/mL	Peprotech	Cat#315-02
Ammonium chloride (NH_4_Cl)	Carl Roth	Cat#9265.1
OVA^257-264^ (1 mg/mL)	InvivoGen	Vac-sin
Pen/Strep (10,000 U/10,000 U)	Thermo Fisher Scientific	Cat#15070-063
RPMI 1640 + L-glutamine	Thermo Fisher Scientific	Cat#21875-091
NaPyr (100 mM)	Thermo Fisher Scientific	Cat#11360-070

**Experimental models: Cell lines**		

PancOVA H_2_B Cerulean	Nasiri et al.^2^ Cell line available through contact with the senior authors.	N/A

**Experimental models: Organisms/strains**		

C57BL/6-Tg(TcraTcrb)1100Mjb/J (OT-I); transgenic T cell receptor; gender irrelevant; age 8 to 16 weeks	The Jackson Laboratory	RRID:003831
OT-Ix*Il18r*^−/−^; IL-18 receptor knockout; transgenic T cell receptor; gender irrelevant; age 8 to 16 weeks	Philipps-University Marburg	N/A
OT-IxTgVenus; lipid-modified YFP, transgenic T cell receptor; gender irrelevant; age 8 to 16 weeks	Philipps University Marburg	N/A
Tg(CAG-Venus)1Hadj/J (TgVenus); lipid-modified YFP; gender irrelevant; age 8 to 16 weeks	The Jackson Laboratory	RRID:011107

**Software and algorithms**		

BioRender	BioRender	https://www.biorender.com
GraphPad Prism 8 9.5.0	GraphPad Software	https://www.graphpad.com
Imaris 9.9	Bitplane-Oxford Instruments	https://imaris.oxinst.com
Inkscape 1.2.1	Inkscape Community	https://inkscape.org
Microsoft Office	Microsoft	N/A

**Other**		

TruLive 3D Imager	Luxendo-Bruker	https://www.bruker.com/en/.html
15 mL tube	Sarstedt AG & Co	Cat#62.554.502
50 mL tube	Greiner Bio-One GmbH	Cat#227261
Cannula 27G Sterican	B. Braun	Cat#4665406
Cell culture plates	Greiner Bio-One GmbH	N/A
Cell strainer 30 μM	Miltenyi Biotec	Cat#130-110-915
Cell strainer 70 μM	Miltenyi Biotec	Cat#130-110-916
Disposable syringe, 1 mL	B. Braun	Cat#9166017V
Forceps	N/A	N/A
Nalgene Rapid-Flow sterile disposable filter unit	Thermo Fisher Scientific	Cat#158-0020
Neubauer counting chamber	Plan Optik AG	Cat#40442702
Scissors	N/A	N/A
TC flask T75	Sarstedt AG & Co	Cat#83.3911.002
TC suspension flask T75	Sarstedt AG & Co	Cat#83.3911.502
TruLive 3D Dishes	ibidi GmbH	Special production by Luxendo

## Materials and equipment

**Table:** T cell and BMDM medium

Reagent	Final concentration	Amount
RPMI 1640 + L-glutamine	N/A	415 mL
FCS	10% (v/v)	50 mL
HEPES (1 M)	3% (v/v)	15 mL
Pen/Strep (10.000 U/mL/10.000 μg/mL)	2% (v/v)	10 mL
NaPyr (100 mM)	2% (v/v)	10 mL
2-mercaptoethanol	0.1% (v/v)	500 μL
**Total**	**N/A**	**500 mL**

Store at 4°C for a maximum of 8 weeks; heat up to 37°C before usage.

**Table:** PancOVA medium

Reagent	Final concentration	Amount
DMEM	N/A	445 mL
FCS	10% (v/v)	50 mL
Pen/Strep (10.000 U/mL/10.000 μg/mL)	1% (v/v)	5 mL
**Total**	**N/A**	**500 mL**

Store at 4°C for a maximum of 8 weeks; heat up to 37°C before usage; add Geneticin selective antibiotic (G418 disulfate solution) to a final concentration of 500 μg/mL to the PancOVA medium.

**Table:** Erythrocyte lysis buffer

Reagent	Final concentration	Amount
NH_4_Cl	155 mM	4.15 g
KCO_3_	10 mM	0.501 g
EDTA	0.1 mM	0.019 g
dH_2_O	N/A	500 mL
**Total**	**N/A**	**500 mL**

Store at 4°C for a maximum of 6 months.

## Step-by-step method details

### Generation of macrophages from murine bone marrow

**Timing: procedure, 60–90 min; proliferation and differentiation, 4 days; start 6 days before co-culture**

The following section provides the collection and differentiation of bone marrow-derived cells into BMDMs.

1. Collection of the femur.
  a. Prepare a 15 mL tube or petri dish.
  b. Sacrifice a TgVenus-positive mouse using an authorized method, disinfect, and immobilize in the supine position.
  c. Wet the cadaver with an alcohol-based solution for disinfection, e.g., 70% (v/v) ethanol.
  d. Using scissors, carefully remove the skin and pull back the entire leg skin to expose the leg.
  e. Remove the connecting tissue between the femur and the hip joint.
  f. Using forceps or hands, gently twist the femur to dislocate the bone and cut the femoral head from the hip with scissors.
  g. Remove major muscles like the quadriceps around the femur with scissors.
  h. Carefully twist the knee joint between femur and tibia using forceps.
  i. Cut the knee joint and remove connective tissue.
  j. Remove excess muscle and connective tissue.
  k. Store bones in 5 mL of ice-cold PBS until transferred to a laminar airflow.
2. Rinsing of bone for bone marrow-derived cells.
  a. Prepare a 50 mL tube (one per mouse).
  b. Rinse the bone marrow from the femurs into the 50 mL tube with HBSS after gently cutting the proximal and distal tips of the bone (known as epiphysis).
    i. Use a 5 mL syringe full of ice-cold HBSS with a 27-gauge needle.
    ii. Insert the needle in the proximal metaphysis and carefully flush. Repeat this step for the distal metaphysis.
    iii. Homogenize the bone marrow cells by gently resuspending the cells in HBSS medium.
  c. Prepare a 50 mL tube, a 1 mL syringe plunger, and a 70 μM cell strainer (one per mouse).
  d. Pass the cell suspension through the cell strainer to remove bone fragments and blood clots. Crush the remaining bone marrow fragments with the flat side of the plunger of the 1 mL syringe.
  e. Rinse the cell strainer with ice-cold HBSS and fill the 50 mL tube up to 20 mL.
  f. To pellet cells, centrifuge the tube at 400 × *g* for 5 min at room temperature (RT; 20°C–25°C).
  g. Discard the supernatant after centrifugation.
  h. Add 5 mL erythrocyte lysis buffer to the cell pellet and resuspend the cells in the buffer.
  i. Incubate for 3 min at RT. Stop the lysis by filling the tube with 15 mL BMDM medium.
  j. Pellet the cells by centrifuging the tube at 400 × *g* for 5 min at RT.
  k. Discard the supernatant after centrifugation.
3. Seeding and differentiation of bone marrow-derived cells.
  a. Resuspend the cell pellet in 10 mL BMDM medium and calculate the cell number by counting the cells using a device or a counting chamber.
    ***Note:*** In Slusny et al., the cell number was calculated using a Neubauer counting chamber.
    ***Note:*** Two femurs from one mouse provide approximately 2–4 × 10^7^ cells.
  b. Adjust the cell number to 1 × 10^6^ cells per mL in BMDM medium.
  c. For 4 wells in a 24-well plate, prepare a 15 mL tube with 4.5 × 10^6^ cells in 4.5 mL BMDM medium.
  d. Add recombinant mouse M-CSF to a final concentration of 50 ng/mL.
  e. Seed 1 mL of cell suspension into the wells of a 24-well plate.
  f. Place the cells in the incubator at 37°C, 5% CO_2_.
  g. Differentiate the cells for 4 days.
    ***Note:*** If a higher cell density is needed, isolate the tibias as well. In our experience, two femurs and two tibias provide approximately 6–12 × 10^7^ cells per mouse. A detailed step-by-step description for the isolation of the tibia can be found in Toda et al.^3^

### Generation of CTLs

**Timing: procedure, 2–2.5 h; proliferation and differentiation, 5 days; start 5 days before co-culture**

In this section, CTLs are generated from the spleen and lymph nodes of OT-I-positive mice. For the spheroid model approach, the T cells are activated in an antigen-dependent manner using OVA^257-264^ (stock: 1 mg/mL).

***Note:*** If you want to use isolated and enriched CD8^+^ T cells and activate the T cells in an antigen-independent manner, we recommend the commercial magnetic bead-based and negative selection MojoSort Mouse CD8 T cell Isolation kit (# 480008) from BioLegend. Alternative kits for T cell enrichment and isolation are also possible. If you use the negative selection approach to enrich T cells, prepare an anti-CD3ε/anti-CD28-coated cell culture plate before starting the isolation.^4^

**Figure 1:**
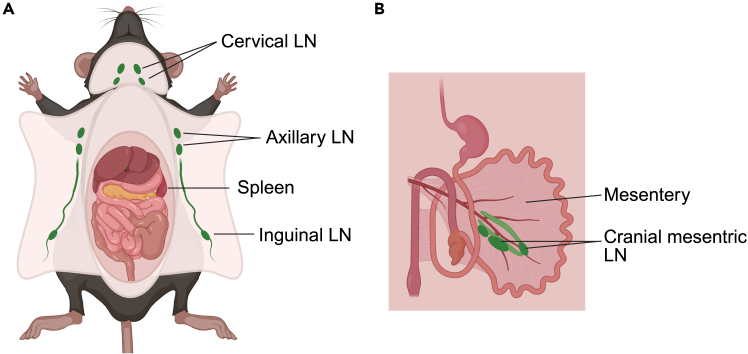
Overview of lymph and spleen collection for isolating T cells Illustration demonstrating the simplified lymphatic collection sites in a mouse. (A) Collection site for the cervical, axillary, and inguinal LN and the spleen. (B) Location of the cranial mesenteric LN in the mesentery. Abbreviation: LN, lymph node. Created in BioRender. Zimmer, V. (2025) https://BioRender.com/cbxldpp.
  d. Collect all lymph nodes in ice-cold HBSS in a 15 mL tube.
  e. Open the abdomen and remove the spleen and cranial mesenteric lymph node (Figure 1).
    i. Pinch the abdominal wall with a forceps and gently cut the abdominal wall with a pair of scissors without damaging the organs in the peritoneal cavity.
    ii. Lightly pull the intestinal organs outside of the peritoneal cavity.
    iii. The cranial mesenteric nodes are along the artery in the mesentery (the tissue that connects the intestine) (Figure 1B).
    iv. The spleen is located on the left side, under the rib cage, and hidden behind the stomach (Figure 1A).
    v. Gently hold the spleen with one forceps and remove the connecting tissue by cutting it with scissors.
  f. Transfer the mesenteric lymph node and spleen to the tube with the previously dissected lymph nodes.

***Note:*** To validate that the extracted nodes are lymph nodes and not fat tissue, observe the lymph nodes in the HBSS solution. Lymph nodes sink. Fat tissue will float on the surface of the solution. Remove the fat tissue.

4. Collection of spleen and lymph nodes.
  a. Sacrifice an OT-I-positive mouse using an authorized method, disinfect it, and immobilize it in the supine position.
  b. Prepare a 15 mL tube with 5 mL ice-cold HBSS (one per mouse).
  c. Dissect axillary, cervical, and inguinal lymph nodes (Figure 1A).
    i. In general, pinch the fascia (the thin membrane covering the fat and tissue) on top of the lymph node with one forceps and pull lightly without breaking the fascia.
    ii. Place the second forceps underneath the lymph node.
    iii. Break the fascia with the first forceps and remove the lymph node by pulling the lymph node lightly.

5. Preparation of single cell suspensions from spleen and lymph nodes.
  a. Prepare a 50 mL tube, a 1 mL syringe plunger, and a 30 μM cell strainer (one per mouse).
  b. Mash the organs with the flat side of a 1 mL syringe plunger through a 30 μM cell strainer with ice-cold HBSS.
    i. Pour the HBSS containing the lymph nodes and the spleen into the cell strainer.
    ii. Use the flat side of the plunger to apply force to the organs and degrade them gently.
    iii. Squash the organs to cell pulp with the plunger and rinse with ice-cold HBSS.
    iv. Repeat the previous step until there is no leftover of the organs on the filter of the cell strainer.
  c. Fill up to 20 mL with HBSS through the cell strainer.
  d. Pellet the cells by centrifuging at 400 × *g* for 5 min and discard the supernatant.
  e. Resuspend the cell pellet in 5 mL of erythrocyte lysis buffer and incubate for 5 min at RT.
  f. Stop the lysis by adding 15 mL T cell medium.
    i. Heat the T cell medium at 37°C before usage.
  g. Pellet the cells by centrifuging at 400 × *g* for 5 min and discard the supernatant.
6. Activation by SIINFEKL.
  a. Resuspend the cells in 20 mL T cell medium and calculate the cell number by counting the cells using a device or a counting chamber.
  b. Prepare a 15 mL tube (one per mouse).
  c. Adjust the cell number to 4 × 10^7^ cells in 1 mL T cell medium, transfer the cells into the 15 mL tube, and add 5 μg/mL of OVA^257–264^ (stock: 1 mg/mL).
  d. Place the tube in the incubator at 37°C, 5% CO_2_ for 1 h.
    i. Don’t tighten the tube lid.
7. Seeding of activated T cells.
  a. Add 9 mL T cell medium to the cells and pellet the cells by centrifuging at 400 × *g* for 5 min.
  b. Discard the supernatant after centrifugation and resuspend the cell pellet in 1 mL T cell medium.
  c. Prepare a T75 suspension flask with 14 mL pre-heated T cell medium.
  d. Count the cells, adjust the cell number to 2–3 × 10^6^ cells/mL, and add 1 mL to the T75 suspension flask.
  e. Add recombinant mouse Interleukin (IL)-12 to a final concentration of 10 ng/mL and incubate the cells for 48 h at 37°C, 5% CO_2_.
8. Proliferation and differentiation of T cells.
  a. After 48 h (day 2 after T cell activation, step 7) and 96 h (day 4 after T cell activation, step 7) of T cell differentiation, provide fresh T cell medium for proliferation.
    i. Heat the T cell medium at 37°C before usage.
    ii. Prepare a 50 mL tube.
    iii. Prepare a T75 suspension flask with 14 mL pre-heated T cell medium.
    iv. Collect the cell suspension in the 50 mL tube and centrifuge at 400 × *g* for 5 min.
    v. Discard the supernatant and resuspend the pellet in 1 mL T cell medium.
    vi. Add the cell suspension to the T75 suspension flask and add recombinant mouse IL-2 at a final concentration of 20 ng/mL.
    vii. Place the T75 suspension flask in the incubator at 37°C, 5% CO_2_.

### Polarization of BMDMs

**Timing: procedure, 5 min; performed at day 4 of BMDM differentiation**

This step describes the polarization of BMDMs into M1- or M2-like macrophages one day before spheroid formation. Carefully check the adhesion and morphology of BMDMs before starting steps 9 to 11. The cells must be attached to the plate bottom and the morphology should be round and oval.

9. Preparation of polarization for BMDMs with an M1-like phenotype.
  a. Heat the BMDM medium at 37°C before usage.
  b. For 2 wells of a 24-well plate, prepare 2.5 mL BMDM medium and add 200 ng/mL LPS and 40 ng/mL recombinant mouse Interferon (IFN)-γ.
    i. This is called the M1-polarizing mix as it induces an M1-like phenotype.
  c. Gently add 1 mL of the M1-polarization mix to each well of the 24-well plate for a final concentration of 100 ng/mL LPS and 20 ng/mL IFN-γ per well.
10. Preparation of polarization for BMDMs with an M2-like phenotype.
  a. Heat the BMDM medium at 37°C.
  b. For 2 wells of a 24-well plate, prepare 2.5 mL BMDM medium with 40 ng/mL recombinant mouse IL-4.
    i. This is called the M2-polarizing mix as it induces an M2-like phenotype.
  c. Gently add 1 mL of the M2-polarization mix to each well of a 24-well plate for a final concentration of 20 ng/mL IL-4 per well.
11. Polarization into M1- or M2-like BMDMs.
  a. Place the cells in the incubator at 37°C, 5% CO_2_.
  b. Polarize for 24 h.

### Generation of spheroids

**Timing: 2–2.5 h; formation, 1 day**

**Timing: 30–45 min (for step 12)**

**Timing: 40–50 min (for step 13)**

**Timing: 50–60 min (for step 14)**

Here, we describe the generation and formation of spheroids in the presence of polarized BMDMs. For the generation of three-dimensional spheroids, Corning Matrigel is used, which serves as a 3D scaffold for the cells in this protocol. Matrigel is an extracellular matrix (ECM)-mimicking hydrogel, composed of hydrophilic polymers that are not cross-linked, thus modeling structures and properties of ECM.^5^^,^^6^

It is essential to control the adhesion of BMDMs before starting the spheroid formation. BMDMs with M1 or M2 phenotype must be attached to the plate bottom. As reported by McWhorter et al., an M1 polarizing mix leads to a round, pancake-like shape, whereas an M2-polarizing stimulus induces an elongated cell shape.^7^

12. Harvest of BMDMs for spheroid formation and FACS analysis.
  a. Heat PBS, BMDM medium, and Accutase at 37°C before usage.
  b. Remove the medium from the cell culture plate and carefully rinse the wells by gently adding 2 mL PBS, carefully swirling the plate, and aspirating the PBS.
  c. Add 300–400 μL Accutase to each well of the cell culture plate.
  d. Incubate the plate at 37°C, 5% CO_2_ for 10–15 min. Check the plate visually for detachment of the cells from the well bottom.
  e. Collect the cell suspension in 1.5 mL reaction tubes by gently pipetting up and down. Check the plate visually for remaining cells.
    ***Optional:*** Add another 500 μL PBS to each well, repeat pipetting up and down, and collect the cell suspension in the same 1.5 mL reaction tube.
  f. Pellet the cell suspension by centrifuging at 400 × *g* for 5 min and discard the supernatant.
  g. Resuspend the cell pellet in 0.5–1 mL BMDM medium and calculate the cell number by counting the cells using a device or a counting chamber.
  h. Set aside 5 × 10^4^ cells in a fresh 1.5 mL reaction tube for spheroid generation.
  i. Store at 37°C, 5% CO_2_ until step 14 (approx. 20–30 min).
    ***Note:*** The cell number of BMDMs for each experimental setup of this assay is 8 × 10^3^ cells. The cell number can also be adjusted; however, titration of the BMDM cell number is necessary beforehand.
    ***Optional:*** We decided to use the remaining BMDMs to validate their respective phenotype via FACS analysis as described in our recent study.^1^
13. Harvest of PancOVA H_2_B Cerulean tumor cells.
  a. Heat the PancOVA medium and trypsin at 37°C before usage.
  b. Prepare a 50 mL tube.
  c. Remove the medium from the PancOVA H_2_B Cerulean cells and gently rinse the cells with PBS by adding PBS to the flask or dish, swirling the flask or dish, and aspirating the PBS.
  d. Add trypsin to detach the cells from the flask or dish and incubate for 2–5 min at 37°C, 5% CO_2_ in the incubator. Check the dish or flask visually for detachment of the cells.
  e. Stop the trypsinization by adding twice as much PancOVA medium as trypsin.
  f. Collect the cell suspension in a 50 mL tube and centrifuge for 5 min at 400 × *g*.
  g. Discard the supernatant and resuspend the cell pellet in 20 mL PancOVA medium.
  h. Repeat the centrifugation of the cells (5 min at 400 × *g*) and discard the supernatant.
  i. Resuspend the cells in 5 mL PancOVA medium.
  j. Calculate the cell number by counting the cells using a counting chamber or device.
  k. Adjust the cell count to 1 × 10^6^ cells/mL in PancOVA medium.

***Note:*** 6 × 10^4^ tumor cells are used for each experimental condition of this assay. This cell number can be changed; however, protocol adjustment and titration of the PancOVA H_2_B Cerulean cell number is required beforehand.

**Figure 2:**
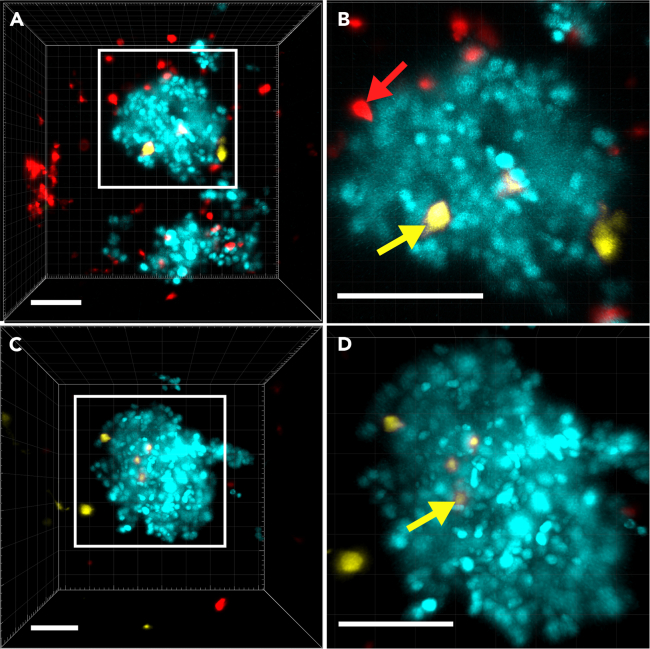
Motility analysis of CTLs in Matrigel spheroids Successful (A and B) and failed (C and D) migration of CTLs in Matrigel after 4 h of co-culture start. CTLs are depicted in red, BMDMs in yellow, and PancOVA cells in turquoise. The white square zooms in for CTLs and BMDMs near a spheroid (B) or not (D). The red arrow points out a CTL near the spheroid surface, and the yellow arrows point out BMDMs near the spheroid surface. The scale bar (white) represents 100 μm.
  j. Start in the corner of the wells to protect the sample and not to destroy the Matrigel.
  k. Place the dishes in the incubator at 37°C, 5% CO_2_, overnight (18–24 h).
    i. Check the formation of the spheroid culture in the presence of BMDMs before proceeding with the harvest of the T cells.
    ii. Samples with small spheroids can be incubated longer (a few hours or overnight) (see “troubleshooting”).
      ***Note:*** The BMDMs are not involved in the generation of spheroids. Only PancOVA H_2_B Cerulean tumor cells form spheroids in the Matrigel.

14. Spheroid formation.
  a. Prepare reaction tubes for each experimental condition that will be measured.
  b. Coat the Ibidi TruLive 3D dishes with 300 μL pre-warmed FCS for 15 to 20 min at RT before adding a mix of PancOVA H_2_B Cerulean cells and BMDMs.
  c. Mix 6 × 10^4^ tumor cells with 3 × 10^3^ BMDMs in one 1.5 mL reaction tube per experimental condition and well.
  d. Centrifuge the cell suspension at 400 × *g* for 5 min at RT.
    i. Check for a cell pellet before proceeding further.
  e. Carefully discard the supernatant and FCS in the Ibidi TruLive 3D dishes.
    ***Note:*** The cell pellet is really small and can easily be overlooked.
  f. Resuspend each cell pellet in 45 μL PancOVA medium and gently add 15 μL Corning Matrigel on ice.
    **Caution:** Avoid bubbles when handling the Matrigel. Otherwise, there will be air bubbles in or on the surface of the Matrigel, which can later influence the resolution using the light-sheet microscope. It is also recommended that a control dish without any BMDMs is prepared.
  g. Carefully pipette the PancOVA-BMDMs-Matrigel mix into the FCS-coated Ibidi TruLive 3D dishes without air bubbles.
    i. For each 1.5 mL reaction tube and, thus, experimental condition prepared, use one well of the Ibidi TruLive 3D dishes.
  h. Gently place the dishes for 15–20 min in the incubator at 37°C, 5% CO_2_ for solidification.
  i. Visually check the wells of the dishes to see if the Matrigel is mostly solidified. If not, incubate further in 5 min intervals.
    ***Note:*** Since the Matrigel is prepared at a ratio of 1:3 in this assay (1 part Matrigel to 3 parts cell suspension), the Matrigel does not become completely solid. In addition, the ratio of Matrigel to cell suspension can vary depending on the Matrigel batch. We suggest that the ratio should be tested and calibrated beforehand. An efficient ratio is determined by the ability not to form a 2D layer but a 3D spheroid-like structure, which enables the migration of CTLs and BMDMs into the tumor spheroids (Figure 2).
    i. Carefully add 300 μL BMDM medium to each well of the Ibidi TruLive 3D dishes.

### Co-culture of CTLs and BMDMs with spheroids

**Timing: procedure, 60–90 min**

**Timing: microscopy, 4 h after co-culture start**

***Optional:*** Extract 200 μL of cell suspension from the CTL flask to analyze the surface marker and cytokine expression by flow cytometry analysis as described in our recent studies.^1^^,^^4^

15. CellTracker staining of CTLs.
  a. Prepare a 15 mL tube.
  b. Heat the CTLs medium at 37°C before usage.
  c. Collect and transfer CTLs into the 15 mL tube.
  d. Centrifuge the cells at 400 × *g* for 5 min.
  e. Discard the supernatant and resuspend the cells in 5 mL ice-cold PBS.
  f. Calculate the cell number by counting the cells using a counting chamber or device.
  g. Adjust the cell number to 2 × 10^6^ cells/mL and transfer 1 mL of the cell suspension into a fresh 15 mL or 2 mL reaction tube.
  h. Centrifuge the cell suspension at 400 × *g* for 5 min.
  i. Discard the supernatant and resuspend the cells in 1 mL ice-cold PBS.
  j. Add 1 μM CellTracker DeepRed (1:1.000 dilution in PBS) to the cell suspension and gently mix before placing the tube for 45 min in the incubator at 37°C, 5% CO_2_.
  k. Centrifuge the stained cells for 5 min at 400 × *g*.
  l. Discard the supernatant and wash the cell pellet by resuspending the cells in 2 mL T cell medium and centrifuging the cell suspension (400 × *g*, 5 min).
  m. Discard the supernatant.
  n. Resuspend the stained cells in T cell medium to a cell number of 1.2 × 10^5^ cells per 300 μL.

***Note:*** Other CellTracker dyes or similarly working dyes can be used for this assay. Also, the dyes must be titrated beforehand to ensure the brightness of the labeling and cross-talk between the used dyes and fluorophores.

16. BMDMs and CTL interaction in the spheroid model.
  a. Carefully remove the medium from Matrigel-samples and gently add 300 μL T cell medium with 1.2 × 10^5^ cells per condition on top of the wells, again starting at the well’s corner (see “troubleshooting”).
  b. Place the TruLive 3D dishes in the incubation chamber of the light-sheet microscope TruLive 3D, set to 37°C, 5% CO_2_.

***Note:*** The dishes can also be stored in an incubator until they can be placed in the microscope's incubation chamber.

***Note:*** Placing the dishes directly into the device ensures no additional movement of the TruLive 3D dishes.

17. Imaging with the light-sheet TruLive 3D imager.
  a. After 4 h of adding CTLs to the spheroid-BMDMs co-culture, start imaging.
    ***Note:*** For this assay, fluorophores with distinct excitation and emission wavelengths were chosen (see Table 1). Using other fluorophore dyes or transgenic mice expressing fluorescent proteins is possible if the microscope has the respective lasers. It is also mandatory to test the fluorophore panel before using it to ensure the distinction of different cell types and the combination of fluorophores, regarding cross-talk and intensity of the dyes and fluorophores.
    **Table 1:** An example panel of fluorophores used to distinguish different cell types

Fluorophore	Laser [nm]	Filter [nm]	Stained structure	Staining protocol
H_2_B Cerulean	445	457–501	Histones of the nucleus of the tumor cells	Transgene
tgVenus (YFP protein)	488	526–564	Plasma membrane	Transgene
CellTracker DeepRed	642	655–704	Cytoplasm of cells	Diluted 1:1000 in PBS, incubated 45 min at 37°C

This example panel shows fluorophores used by Slusny et al.^1^
  b. Screen for spheroids using transmitted light and mark them.
    i. An optimal spheroid has a diameter of about 100–250 μm, is three-dimensional, and almost round (Figure 2).
    ii. It is crucial that the software saves the position of the regions so that the marked areas can be found again later.
  c. Select as many regions as possible along the length of the sample holder (x-axis) and the width of the v-shaped chamber (y-axis) (5–20 per well or condition).
    i. Give each area within a well a designated number to correctly identify them.
    ii. The entire length and width of the wells of the TruLive 3D dishes should be used.
    iii. There should be at least 5 spheroids per well. Otherwise, the formation of the spheroids was not successful, or the spheroids were too small when CTLs were added.
  d. After marking a position, every spheroid’s z-position and z-distance are defined.
    i. By defining each spheroid’s bottom and top sides, the z-distance between two z-layers is set to 3 μm.
  e. Check for the infiltration of CTLs in the Matrigel near the spheroids (Figure 2).
    i. This is done with the corresponding lasers and filters (see Table 1).
    ii. The ratio of Matrigel-infiltrating CTLs to tumor cells should be around 1:8 or 1:10.
    iii. If less than a handful of CTLs have migrated into the Matrigel at the time of measurement (4 h after the start of the co-culture), leave the co-culture for another 1–2 h and check again (see “troubleshooting”).
  f. Ensure the presence of BMDMs in the spheroid regions.
    i. When mixed with them, the BMDMs should be deployed in a ratio of 10:1 (ten BMDMs to one tumor spheroid) (see “troubleshooting”).
    ii. The absence of BMDMs suggests an error occurred during the mixing and pipetting of the BMDMs-PancOVA-Matrigel mix.
  g. Depending on the presence of CTLs and BMDMs near the spheroids, select 2 spheroid regions for imaging.
    ***Note:*** It is also possible to select more regions per well; however, the recording and duration of one-time intervals for every region will take longer. We recommend two areas of two wells or conditions for one recording to keep the one-time interval 60–90 s long.
    ***Note:*** A video recording of approximately 30 min (one-time interval of the selected areas every 60 s) is preferred for migration pattern and movement analysis.
    ***Note:*** Selecting two or more spheroid areas per experimental condition serves as technical controls and replicates. It is recommended to select at least two, as sometimes spheroids migrate out of the marked region.
  h. Start the recording and check how long the one-time intervals of the selected areas take.
    i. The default channel’s settings for the TruLive Imager are typically 20–50 ms of exposure time, a laser power of 10 to 20%, with only 1% of laser power coming through the attenuation filter. These settings were also used in Slusny et al.^1^
    ii. The device has to move according to the appointed settings along the x- and y-axis and the z-stacks.
  i. Repeat the last step for all conditions and wells. Each recording takes 30–45 min.

### Tumor disintegration analysis

**Timing: undefined, how long imaging takes**

18. Tracking of spheroid deformation overnight.
  a. After the 30 min long videos of the spheroids, select the same areas (or more) and start a time-lapse video that includes all regions and z-stacks with a time interval of every 60 min.
    ***Note:*** It is also possible to shorten the interval to 20 or 30 min.
    ***Note:*** Consider that the data collected is enormous if the recording interval is small (sCMOS camera images with 2048 × 2048 pixels).
  b. After 18 h of starting the co-culture by adding CTLs to the BMDM-spheroid culture and after recording overnight, start another 30 min long video.
    i. Beforehand, check the condition of the spheroids (also see “troubleshooting”).
    ii. The second time point allows a comparison of motility and cell movement to the beginning of the co-culture.

## Expected outcomes

The spheroid assay described in this protocol generates a very high data volume. First and foremost, there are the images and videos of the spheroids themselves. Moreover, the outcome includes various parameters from quantity, volume, morphology, and motility analysis of three different cell types and their interactions. Previously published results and figures provide examples of data from our recent study.^1^

## Quantification and statistical analysis

### Microscopy analysis using Imaris

During the recording time of the spheroid co-culture (steps 17 and 18), videos of chosen areas from every well in at least two duplicates were acquired. Staining the CTLs with the CellTracker DeepRed and isolating macrophages from the bone marrow of tgVenus-positive mice allowed the discrimination of the two populations and analysis of their motility. In the processing tool of the Luxendo software, data were converted into the Imaris format and reduced by a factor of 9 and by 3 × 3 voxel binning in xy to reduce pixel size and, with this, the size of the Imaris file. The analysis was done using the Imaris 9.9 software.

### Detection of tumor spheroids

**Figure 3:**
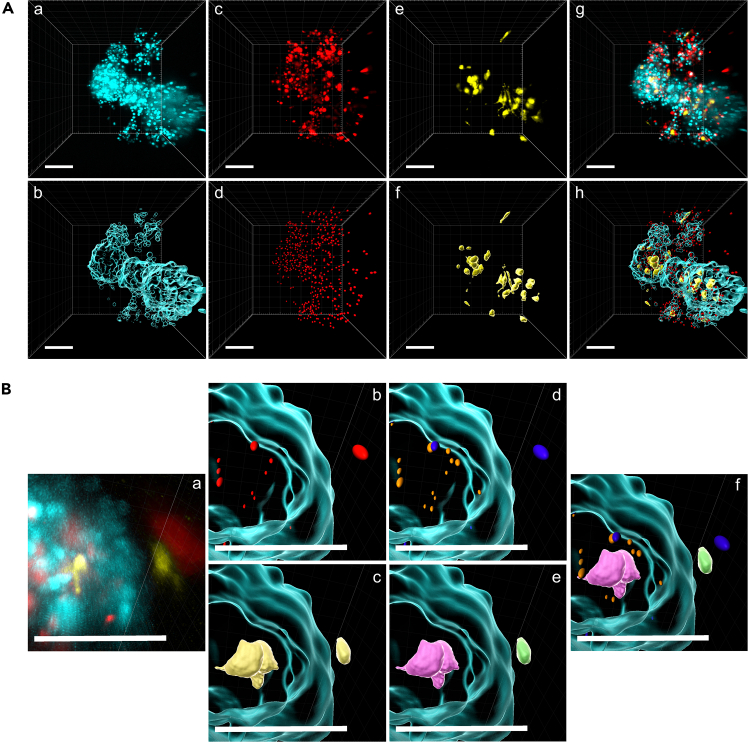
Application of the surface and spot algorithm (A) Applying the surface algorithm (b) on PancOVA spheroids (a, turquoise). For the CTLs (c, red), the spot algorithm is chosen (d), while the surface algorithm (f) is also used for macrophages (e, yellow) due to their irregular shape. Merged channels without (g) or with respective applied algorithms (h). (B) Merged and zoomed-in picture of a spheroid without algorithm masks (a). Spot (b, red) and surface (c, yellow) algorithms on CTLs or BMDMs. Discrimination of infiltrating (orange) and peripheral (blue) CTLs (d) and infiltrating (purple) and peripheral (green) BMDMs (e). Merged channels with respective algorithm mask. The scale bar (white) represents 100 μm.
• When necessary, a drift correction is done by the index “edit tracks” (correct image and all objects, translational and rotational drift, and include all results).
• Volume and sphericity are selected as statistical parameters to export as an Excel file.
• With the index “edit”, the spheroid’s surface is masked to discriminate CTLs and BMDMs inside and outside the spheroid (Figure 3B).
• Using the index “mask all”, select either the channel for CTLs or the channel for BMDMs in the channel selection. Set the “voxel outside” to 0 = infiltrating CTLs/BMDMs and the “voxel inside” to 0 = peripheral CTL/BMDMs.

• Choose the surface algorithm with object-object statistics.
• Select the respective channel (with the dyes in this setup, the turquoise channel), a surface grain size of 3 μm, and absolute intensity. The high grain size is necessary to cover the whole spheroid and not single tumor cells.
• After choosing an intensity threshold, surfaces of spheroids are generated (Figure 3A).

### Detection of CTLs

• As most CTLs are more or less round, choose the spots algorithm with object-object statistics and tracking (Figure 3A).
• The algorithm is the same for infiltrating and peripheral CTLs.
• The respective channel is selected (red in this setup) and background subtraction with 6.24 μm in xy and 12.5 μm in z.
• After defining the quality (intensity and structure of the cell), cells are tracked over time with Brownian movement at a maximal distance of 18 μm, a gap of 1, and a track duration of over 180 s (or over 3 time points).
• Parameters for CTL motility analysis are track displacement length, track duration, track length, track speed mean, instantaneous velocity, and displacement delta length.

### Detection of BMDMs

• As BMDMs have elongations and with this an irregular cell shape, choose the surface algorithm with tracking and object-object statistics (Figure 3A).
• The algorithm is the same for the infiltrating and peripheral BMDMs.
• The respective channel (yellow for TgVenus BMDMs) and absolute intensity are selected.
• For tracking BMDMs, set Brownian motion to a maximal distance of 16 μm, a gap size of 1, and a track duration of over 180 s.
• Parameters for BMDM analysis are track displacement length, track duration, track length, and track speed mean.

### Further analysis

Use the exported Excel files to generate graphs in GraphPad Prism or further analyze them with R Studio. Arrest duration and arrest coefficient are calculated with the instantaneous velocity/ displacement delta length.

## Limitations

The Matrigel-based spheroid co-culture with BMDMs and CTLs allows cell-cell interaction analysis of various parameters of interest. However, the model simplifies the tumor microenvironment as it lacks interaction with further cell populations, cytokines, and chemokines that enable the immunosuppressive tumor milieu.

The protocols were established using mouse T cells from OT-I-positive mice. CD8^+^ T cells from OT-I mice express a transgenic T cell receptor specific to the OVA-peptide SIINFEKL, thus allowing antigen-dependent T cell analysis. However, adjustments might be needed using T cells from wild-type C57BL/6j mice. The migration and motility patterns of these T cells might be different.

Additionally, the protocols were established with mouse pancreatic cell lines Panc02, PancOVA, and T11 that were stably transduced with the plasmid pCS-H_2_B-Cerulean (#53748, Addgene). We noticed differences in the formation of spheroids between cells with a Panc02 background and T11 cells. Thus, adjustments might be needed, e.g., using a breast cancer or lung carcinoma cell line. The size, morphological structure, and formation of spheroids can differ between different disease models.

Moreover, the results of many experiments depend on the quantity of infiltrating CTLs. Their number may vary between experiments due to multiple factors like mouse age or dilution of the Matrigel.

The analysis mentioned above for observing the spheroids, CTLs, and BMDMs bears certain limitations, too. Due to the microscope’s incubation chamber, we can perform long-term recordings of the cells. However, due to varying spheroid size, T cell activity, and limited control of cell–cell interactions, it is difficult to pinpoint the exact time frame for the cytotoxicity-induced deformation of spheroids for every experiment.

## Troubleshooting

### Problem 1

Only few T cells can be found after adding T cells to the spheroid model (Figure 2) (Steps 15–16 in “co-culture of T cells and BMDMs with spheroids”).

### Potential solution

Co-culture longer before imaging, or ensure that a pellet can be seen after the centrifugation step during the staining of T cells. Also, check for cells on the dishes before incubation. Another reason could be that the Matrigel is concentrated too highly. We observed slight but relevant differences between Matrigel batches. The limitations of Matrigel were exceptionally reviewed in Aisenbrey and Murphy.^8^ Accordingly, you may need to titrate the perfect dilution for your experiments.

### Problem 2

Neither spheroids nor macrophages can be found during imaging (Steps 16–17 in “co-culture of T cells and BMDMs with spheroids”).

### Potential solution

The medium of the macrophage-spheroid culture has to be removed carefully. Removal of the medium can lead to the Matrigel being extracted together with the medium. It is recommended that the medium is removed with great caution using a pipette.

### Problem 3

Cells do not form or only form small spheroids (Steps 13–14 in “generation of spheroids”).

### Potential solution

In the case of small spheroids, give the spheroids more time to form. Also, check the passage of your cells or test for mycoplasma, as contaminated and more passaged cells can be slower in proliferation and potentially form fewer spheroids.

### Problem 4

Spheroid disintegration is incomplete (Step 18 in “tumor disintegration analysis”).

### Potential solution

Dissolution of tumor spheroids is associated with quantity, activity, and motility of co-cultured T cells.^1^ Macrophage and spheroid co-culture alone does not result in spheroid disintegration.

## Resource availability

### Lead contact

Further information and requests for resources and reagents should be directed to and will be fulfilled by the lead contact, Christian Bauer (bauerch4@staff.uni-marburg.de).

### Technical contact

Technical questions on executing this protocol should be directed to and will be answered by the technical contact, Katrin Roth (katrin.roth@imt.uni-marburg.de).

### Materials availability

This study did not generate new materials or reagents. All materials mentioned earlier are commercially available, except for the OT-Ix*Il18r*^−/−^ and OT-IxTgVenus mice strains, the PancOVA H_2_B Cerulean cells, and the TruLive 3D dishes. Cell and mouse strains are available through the senior authors.

### Data and code availability

All original analysis code has been deposited at the Zenodo repository (https://doi.org/10.5281/zenodo.15336457).

## Acknowledgments

This research was funded by the 10.13039/501100001659Deutsche Forschungsgemeinschaft (DFG) grants KFO325 BA 3824/3-1, BA 3824/3-2, RO 5599/1-1, and RO 5599/1-2. We thank Bettina Geisel for her technical support. Parts of the graphical abstract were generated using BioRender.com.

## Author contributions

V.Z. and K.R. developed the co-culture and spheroid protocol. V.Z., B.S., and K.R. performed the experiments, data acquisition, and data interpretation. V.Z. and K.R. wrote this protocol. C.B. supervised the study and revised and proofread the manuscript.

## Declaration of interests

The authors declare no competing interests.
